# Visualizing Bacterial Colony Morphologies Using Time-Lapse Imaging Chamber MOCHA

**DOI:** 10.1128/JB.00413-17

**Published:** 2017-12-20

**Authors:** Maria Peñil Cobo, Silvia Libro, Nils Marechal, David D'Entremont, David Peñil Cobo, Mehmet Berkmen

**Affiliations:** a Independent researcher, Beverly, Massachusetts, USA; b New England Biolabs, Ipswich, Massachusetts, USA; c Independent researcher, Torrelavega, Cantabria, Spain; Princeton University

**Keywords:** MOCHA, colony phenotype, microbial movies, time-lapse movie

## Abstract

Capturing microbial growth on a macroscopic scale is of great importance to further our understanding of microbial life. However, methods for imaging microbial life on a scale of millimeters to centimeters are often limited by designs that have poor environmental control, resulting in dehydration of the agar plate within just a few days. Here, we created MOCHA (microbial chamber), a simple but effective chamber that allows users to study microbial growth for extended periods (weeks) in a stable environment. Agar hydration is maintained with a double-decker design, in which two glass petri dishes are connected by a wick, allowing the lower plate to keep the upper plate hydrated. This flexible chamber allows the observation of a variety of microbiological phenomena, such as the growth and development of single bacterial and fungal colonies, interspecies interactions, swarming motility, and pellicle formation.

**IMPORTANCE** Detailed study of microbial life on the colony scale of millimeters to centimeters has been lagging considerably behind microscopic inspection of microbes. One major reason for this is the lack of inexpensive instrumentation that can reproducibly capture images in a controlled environment. In this study, we present the design and use of a unique chamber that was used to produce several time-lapse movies that aimed to capture the diversity of microbial colony phenotypes over long periods.

## INTRODUCTION

The visualization of microbial life began with the birth of the microscope via the seminal work of Robert Hooke (including Micrographia, published in 1665) (1) and Anton van Leeuwenhoek (in 1676) (2). Since then, great progress has been achieved in the microscopic inspection of single-celled life, increasing our capacity to visualize cells to 20-nm resolution using superresolution microscopy (3). Despite this progress, capturing with time-lapse photography the diversity and dynamic biology of microbial interactions at the macroscale of millimeters to centimeters has lagged considerably.

A majority of the studies of microbial communities on the colony scale are conducted using standard cameras placed within incubators (4). One example is the large microbial evolution and growth arena (MEGA) plate, which is used to monitor emerging bacterial mutants in response to increasing levels of antibiotics on a large agar plate (5). Although this method captures microbial growth over time, it is not temperature controlled, it can support colonies for only 12 days, and it has limited resolution for discerning details of colony morphologies, due to the large size of the agar plate (120 cm long). Such experimental configurations usually consist of a computer-controlled digital single-lens reflex (DSLR) camera on a tripod and a light source, facing down onto an agar plate with bacteria, assembled in an incubator.

Although this kind of apparatus can be sufficient for simple and short time-lapse movies of bacterial growth on plates over the course of a few days, it has several significant limitations. One limitation is that standard laboratory incubators lack appropriate ports for data and power cables for the camera, lights, and other equipment. Another significant limitation is the dehydration of the agar plate in the incubator. During incubation of bacteria on agar, water evaporates from the medium, which can result in dehydration and undesirable condensation of the liquid on the lid or even on electronics. This issue can be ameliorated by incubating agar plates upside down, sealed in Parafilm. To avoid condensation on an upright petri dish lid, however, a glass plate must be placed on top of the petri dish. When left in an incubator at >30°C, agar dehydrates rapidly, limiting time-lapse photography to 2 to 4 days.

To overcome these limitations, we built a specialized chamber for capturing, using time-lapse photography, bacterial growth at the colony level in a petri dish. The chamber was designed based on iterative trial and error, resulting in the final design that we have named MOCHA (microbial chamber). Here, we describe a method to build and to use a large-scale chamber and a double-decker petri dish apparatus that can be used to capture microbial growth using a standard camera, computer, and equipment. This simple-to-assemble chamber permits users to automatically take photos of microbial growth on petri dishes at various magnifications and intervals for different periods. Our assembly employs a lower petri dish that has a water reservoir, which feeds the upper agar petri dish with water through a paper wick. With a humidifier placed within the chamber, this design allows time-lapse photography of bacterial growth over periods of more than 40 days. We show examples of the utility of this chamber with time-lapse movies of microbial organisms forming intricate morphologies when grown on solid agar or at the liquid-air interface of a pellicle. Other examples include movies of microbial interactions and fungal growth. We hope that the dissemination of such a design will facilitate the evolution of improved apparatuses for capturing novel microbial phenotypes on the macroscopic scale.

## RESULTS

### System design and demonstration.

**FIG 1:**
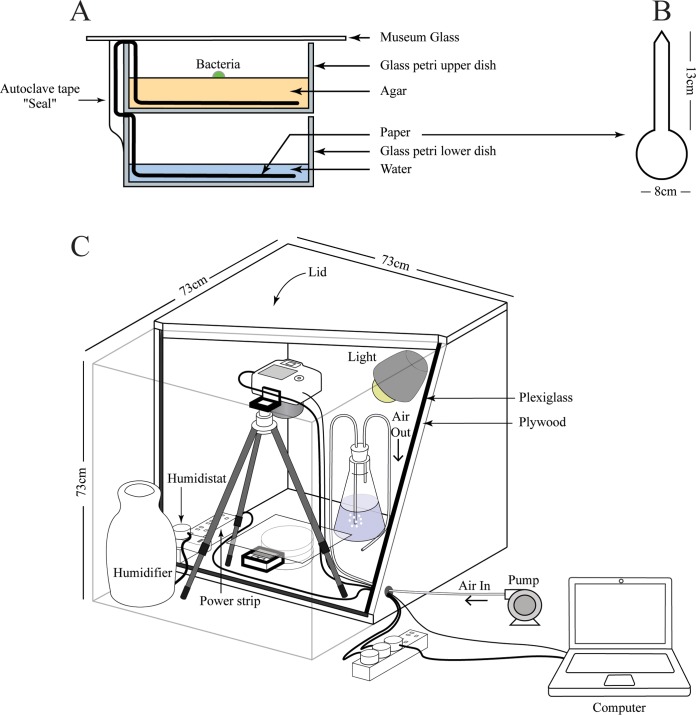
Schematic representation of MOCHA. (A) The double-decker chamber consists of two glass petri dishes stacked on top of each other and is sealed with museum glass. The upper chamber, containing the agar and bacteria, is fed water from the lower chamber with a paper wick. (B) The dimensions of the paper wick are indicated. (C) The double-decker chamber is placed within MOCHA, which houses a humidifier controlled by a humidistat and an air pump connected to a sparger for gas dispersion. The camera is controlled by a computer connected via cables that run through ports.

Microbes display a staggering variety of growth phenotypes on the macroscopic scale, including sectoring, swarming, biofilm formation, pellicle formation, and changes in morphology due to chemical or environmental signaling. These changes usually occur on a time scale of days or even weeks. To capture these diverse morphological changes, we created a specifically designed chamber with controlled humidity that allows observation for >30 days (Fig. 1). Furthermore, we enhanced the capacity of the agar to remain moist with a novel double-decker design, as described in Materials and Methods. A computer-controlled digital camera was integrated into the system so that photographs and time-lapse movies could be obtained. In order to demonstrate the improvements gained with the use of MOCHA, two separate time-lapse movies were obtained. In the first movie, strains of Arthrobacter agilis, Nesterenkonia spp., Bacillus pumilus, and Deinococcus radiodurans were inoculated clockwise starting from the top left corner (see Fig. S1A and Movie S9, first 30 s, in the supplemental material). Due to the lack of hydration, the agar plate started to dry within 48 h and was completely dry within 4 days. Under these conditions, the inoculated bacteria did not develop their natural colors. Unlike the standard agar plates, the double-decker plates inoculated with the same bacteria remained moist throughout the movie, lasting at least 5 days, during which the bacteria were able to develop their natural colors (Fig. S1B and Movie S9, second 30 s).

### Bacillus blooming tree.

**FIG 2:**
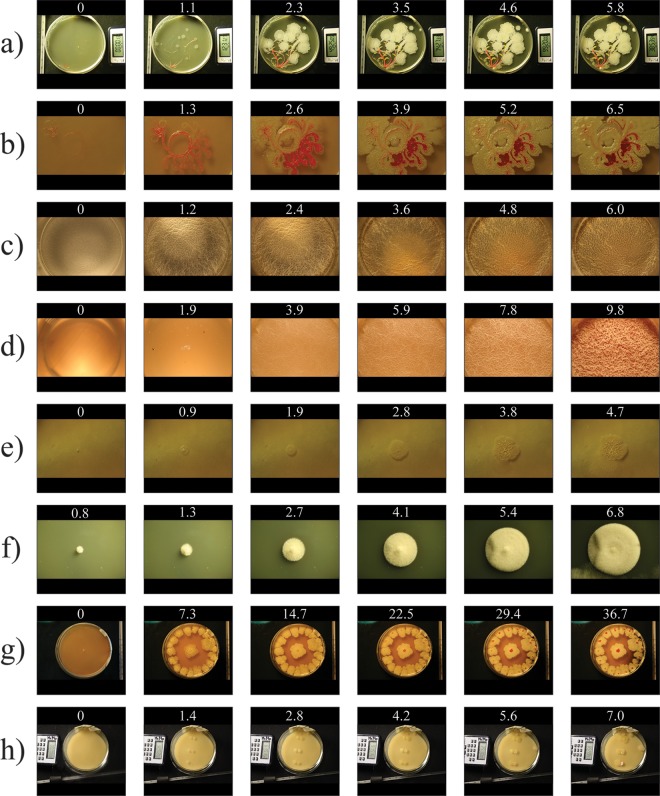
Photographs of microbial growth in MOCHA over time. Representative photos of various types of microbial growth in the MOCHA chamber are shown in a series of photos obtained at six equal time points. The time is shown, as number of days, above each photograph. (a) Bacillus blooming tree. (b) Bacillus-Serratia flower. (c) Bacillus pellicle in a well. (d) Bacillus pellicle in a beaker. (e) Bacillus colony. (f) Fungal colony. (g) Concentric Bacillus colonies with red center. (h) Streptomyces interactions, with colonies of S. coelicolor spotted on the left and colonies of S. viridochromogenes (top), S. albus (middle), and Streptomyces SPB74 (bottom) spotted on the right.

 Certain microbial growth characteristics, such as speed and timing, are not evident in their entirety when shown in a series of photographs. Eight diverse examples are shown as series of photographs in Fig. 2. Compared to the respective time-lapse movies, simple series of photographs reveal a significant loss of visual information. This issue is perhaps best exemplified in the artistic rendition of a “bacterial tree” (Fig. 2a and Movie S1); the movie was recognized as a 2015 winner of the Federation of American Societies for Experimental Biology (FASEB) BioArt competition. The “stems” of the bacterial tree were streaks of the red carotenoid-producing bacterium Arthrobacter agilis, while the “leaves” were streaks of the yellow bacterium Nesterenkonia sp. The fast-growing Bacillus subtilis E42 strain was used for the “flowers” and reached full growth 10 h after inoculation.

### Bacillus-Serratia flower.

Certain colony phenotypes, such as cocultured microbial interactions, need to be observed on a magnified scale to discern subtle changes in colony texture, color, and colony morphology. These subtle changes, when captured in detail, can be indicative of various biological phenomena, such as antibiotic resistance, microbial communications, and evolution within a microcolony (6). In an artistic rendition of a flower, the microbial interactions between Bacillus and Serratia were captured in detail using a Canon EF 100-mm macrolens, which highlights these subtle changes (Fig. 2b and Movie S2). In the time-lapse movie, the Serratia (red colonies) initially outgrows the Bacillus (white colonies) and apparently grows above the Bacillus (the first 8 s of the movie represent 41 h of incubation) but later is outgrown by the Bacillus, which surrounds the Serratia and eventually engulfs it.

### Bacillus pellicle formation in a well.

The molecular mechanisms underlying the formation of pellicles, the exquisite biofilms at the air-liquid interface, have been studied extensively (7). We modified our time-lapse movie methodology to capture details of pellicle formation on the macroscopic scale. In this example (Fig. 2c and Movie S3), the double-decker chamber was replaced by a single well from a 12-well tissue culture plate, which was filled with 4 ml of minimal salts-glutamate-glycerol (MSgg) medium and inoculated with an overnight culture of Bacillus subtilis E42. Rapid growth of cells was observed within the first 24 h and was followed by the formation of the pellicle within the next 6 h. The growth of the pellicle was monitored for the next 6 days, and no significant change was observed. With the appropriate modifications, MOCHA represents a versatile system that should permit detailed characterization of pellicle formation in a controlled reproducible environment, as many mechanistic details of pellicle formation remain to be elucidated (8).

### Bacillus pellicle formation in a beaker.

To observe pellicle formation in more detail, we used a 500-ml glass beaker filled with 250 ml MSgg medium and inoculated with an overnight culture of B. subtilis E42. To avoid condensation from the evaporating liquid medium, the museum glass was omitted. Due to the higher dilution of the overnight culture, slower initial growth was observed (Fig. 2d and Movie S4), compared to the pellicle formation in a tissue culture well. Intriguingly, with the beaker being open to the air, Bacillus growing on the pellicle medium seemed to attract flies, which never appear except in experiments in which Bacillus is grown. Most interestingly, prior to the formation of the pellicle (Movie S4, 40 to 50 s), features present on the surface of the medium are displaced toward the outer edge of the beaker. This could be due to the production of a burst of surfactin, which is known to be important for pellicle formation (9). This movie highlights the necessity of both the timing and regulation of gene expression, which is integral to the formation of a robust pellicle. By setting appropriate time-lapse intervals, MOCHA's versatility allows researchers to capture time-specific patterns, which can be missed when serial photos obtained at fixed long intervals are used.

### Bacillus colony formation.

We inspected a single Bacillus colony in detail, using a macrolens, to assess whether MOCHA can be used to reveal further morphological details in the formation of a single colony. An overnight culture of B. subtilis E42 was inoculated onto rich agar using the double-decker chamber, and a time-lapse movie was recorded over the course of 5 days (Fig. 2e and Movie S5). Interestingly, a sudden outgrowth from the colony was captured toward the end of the movie (Movie S5, 19 to 23 s). This could be due to a change in gene expression that affects the release of extracellular DNA (eDNA). Lysis-independent release of genomic DNA has been shown to play a significant role in the formation of extracellular matrix, facilitating the formation of a robust biofilm (10). Naturally occurring mutants of Bacillus that lack the capacity to release eDNA arise during biofilm formation, which results in a weaker matrix and thus permits outgrowth away from the biofilm.

### Fungal colony.

To exemplify the use of our chamber for the macroscale growth of a eukaryote, a time-lapse movie of a single fungus grown on a rich plate was captured using our apparatus (Fig. 2f and Movie S6). Fine details of hyphae can be observed during the growth of the colony.

### Bacillus displaying novel colony phenotype.

This colony was first isolated after artist Maria Peñil Cobo kissed an agar plate (Fig. S2). We confirmed that the bacterial colonies were Bacillus amyloliquefaciens by sequencing the 16S rRNA using the primers 16S-f (AGAGTTTGATCMTGGCTCAG) and 16S-r (AAGGAGGTGATCCANCCRCA). This simple time-lapse movie of concentric colonies revealed the appearance of a red center only ∼20 days after inoculation (Fig. 2g and Movie S7). This phenomenon has thus far not been observed (R. Losick and R. Kolter, personal communication) and may reveal a novel response to long-term growth. The capacity for long-term microbial growth in MOCHA has captured this potentially novel phenotype.

### Streptomyces interactions.

Many bacteria, such as Streptomyces species, communicate via chemical signals. For Streptomyces, the communication can result in various morphological changes such as changes in colony color (4). To capture these interactions, an overnight culture of Streptomyces coelicolor (left colonies) was spotted on the double-decker petri dish next to a spot of Streptomyces viridochromogenes (top right colony), Streptomyces albus (middle right colony), or Streptomyces SPB74 (bottom right colony) (Fig. 2h and Movie S8). The formation of pink color in the colony of Streptomyces SPB74 on the side interacting with S. coelicolor was visible 3 days after inoculation.

## DISCUSSION

The importance of studying colony morphologies has been long recognized (11, 12). Studying microbial life on solid media through time-lapse movies can capture events that are subtle and occur slowly over days and sometimes weeks. One reason for the lack of centimeter-scale studies of microbial colonies over long periods is the lack of reliable and inexpensive methods to capture microbial colony morphologies on a petri dish. An important factor in consistently obtaining reproducible observations on agar plates is maintaining a controlled environment in which the agar medium does not dehydrate. This is especially important when surface tension is a key factor, such as when studying fractal swarming patterns in Paenibacillus. Although much is known about its fractal-forming morphology on the microscopic scale (13), little is known of the factors governing its macroscopic morphology (14). The MOCHA apparatus we describe here allows such studies to be carried out.

The most important advance in our apparatus is the double-decker petri dish, in which the lower chamber supplies water to the agar in the upper chamber. Although in these incubations we used the lower chamber only to supply water to the upper chamber, chemical inducers such as isopropyl-β-d-thiogalactopyranoside (IPTG), amino acids, or signaling molecules that either are volatile or may be consumed by bacteria during growth could easily be included; this would allow delivery of a constant supply of known amounts of nutrients and other chemicals to the upper petri dish. This feature, along with the special chamber we designed, permits the capture of microbial growth on a petri dish for >40 days, without dehydration of the agar.

One limitation of our design is random contamination (usually fungal contamination), which appears 1 or 2 weeks after inoculation. We suspect this is due to long incubation periods at high humidity. The addition of antifungal chemicals, such as cycloheximide (product no. C7698; Sigma) or amphotericin B (product no. A2942; Sigma), may resolve this problem.

Using MOCHA, we were able to capture various microbial colony phenotypes and their intrinsic beauty, which are difficult to capture with a series of static photos representing the colony morphologies over time (15). This can be clearly seen in our Bacillus blooming tree time-lapse movie (see Movie S1 in the supplemental material). This movie demonstrates that, while time-lapse movies can be used to study colony growth in the laboratory, artistic time-lapse movies of bacteria created with our apparatus can be an effective tool in connecting with the larger nonmicrobiologist community. The beauty of these movies could potentially lessen the exaggerated fear of microbes in our society and thus promote interest in the microbial world, leading to a deeper understanding of both beneficial and harmful microorganisms. The artistic work we performed with our time-lapse apparatus won the annual BioArt competition, organized by FASEB, in 2015 (16).

In addition to demonstrating the aesthetic qualities of bacteria, MOCHA can be used to inspect microbial colony phenotypes for scientific purposes, including the examples of the growth of Bacillus and a fungus, the formation of a Bacillus pellicle, and the interaction of Streptomyces species presented in this work. These studies have revealed emerging Bacillus mutants whose siblings escape the colony, as well as changes in Streptomyces colony color due to chemical communication with neighboring cells (4). Intriguingly, it was observed that flies were attracted to the pellicle, which suggests that the pellicle may contain a chemical attractant (17, 18).

To expand the quantification of the data collected, we introduced a timer and a thermometer, which appeared in some of our time-lapse movies. Additionally, thermoregulation could be improved; currently, the internal temperature of the chamber fluctuates between 22°C and 25°C over 24 h. The addition of a small thermostat could easily permit regulation of the chamber temperature and increase control of the environment. Other metrics and regulators could easily be included, such as controllers for humidity, light, and O_2_/CO_2_ contents, which would allow regulation light exposure and gas composition and provide additional compounds to the agar plate.

Although MOCHA will be useful to many microbiologists for inspection of microbial life in a petri dish in the laboratory, progress is needed for *in situ* visualization of microbial mats and biofilms in their native habitats. This may perhaps be achieved with an open-bottom MOCHA-like chamber to be placed on top of the actual environment, to make movies *in situ*. We sincerely expect MOCHA not only to expand the understanding of microbial life on a scale of millimeters to centimeters but also to increase public awareness of microbial life through beautiful time-lapse movies and to give rise to new and improved designs that can be achieved only with the dissemination of ideas.

## MATERIALS AND METHODS

### Strains and media.

The bacterial strains used in this work were Streptomyces coelicolor, Streptomyces viridochromogenes, Streptomyces albus, Streptomyces SPB74, Bacillus subtilis E42, Serratia marcescens, Arthrobacter agilis, and Nesterenkonia sp. The solid medium used for the growth of bacteria was agar with rich medium (10 g tryptone, 5 g yeast extract, 5 g NaCl, and 15 g agar, with NaOH to pH 7.2, in 1 liter of water) or, for Streptomyces, International Streptomyces Project 2 (ISP2) medium (4 g yeast extract, 4 g glucose, 10 g malt extract, and 15 g agar, in 1 liter of water) (product no. DF0770-17-9; Fisher Scientific). Pellicles were formed in MSgg liquid broth (5 mM potassium phosphate [pH 7], 100 mM morpholinepropanesulfonic acid [MOPS] [pH 7.0], 2 mM MgCl_2_, 700 μM CaCl_2_, 50 μM MnCl_2_, 50 μM FeCl_3_, 1 μM ZnCl_2_, 2 μM thiamine, 0.5% glycerol, 0.5% glutamate, 50 μg/ml tryptophan, and 50 μg/ml phenylalanine) (9), either in a beaker or in a 12-well tissue culture plate (product no. 353043; Falco).

### Double-decker petri dishes.

Two Pyrex petri dishes (product no. UX-34551-05; Cole-Parmer) were stacked on top of each other, as shown in Fig. 1A. The upper dish acted as the lid to the lower chamber. A paper wick, with an 8-cm-diameter circle and a 13-cm wick, was cut from Whatman paper (product no. 10 426 994; GE Healthcare Life Sciences) (Fig. 1B). The disk part of the wick was placed in the upper chamber, and the paper wick was bent such that it entered the lower chamber. The portion of the paper wick running outside the plates was covered with a piece of aluminum foil (approximately 4 cm by 4 cm), and the edges were sealed with autoclave tape (length, 4 cm). To avoid contamination, the paper was placed against the glass as tightly as possible and the aluminum foil was positioned such that it was covering the paper wick completely and did not touch the inside of the lower chamber. Tape and foil were used to reach above the inner edge of the plates and did not touch the agar (Fig. 1A). This arrangement permitted the agar in the upper chamber to be fed with water through the connecting paper wick. The double-decker chamber with the sealed paper wick was autoclaved at 121°C for 20 min and stored at −20°C until use. The molten agar-based medium was allowed to cool and then was poured into the upper deck of the cold double-decker chamber removed from the freezer. This method expedited the solidifying of the agar and prevented the agar from diffusing through the paper wick back into the lower chamber. The double-decker chamber was then allowed to dry at room temperature for 4 days or until a weight loss of approximately 10% occurred. Prior to inoculation, 5 ml of sterile water was pipetted into the lower chamber, using sterile technique. The agar in the upper chamber was inoculated with the desired microorganism(s), and the lid was replaced with a slide of museum glass (Fine Finishes Art and Frame, Ipswich, MA) that had been prewashed with ethanol. The museum glass reflects less light than normal glass and was necessary to limit reflections.

### Time-lapse chamber.

A square chamber (73 cm in length and width) was constructed using plywood (Fig. 1C). The inside of the chamber was lined with Plexiglas and sealed to limit water evaporation. A handle was attached to the upper lid, to facilitate opening and closing of the chamber. The inoculated double-decker petri dish was placed in the middle of the chamber on top of black cloth. Optional equipment, such as a thermometer and a timer, can be placed beside the double-decker chamber to monitor temperature and time, respectively. The camera, on a tripod, was set on top of the double-decker chamber, facing directly down into the chamber. The camera was connected to a power supply and was remotely controlled by a computer. A humidifier (Hunter 31008 Microban) was connected to a humidistat (Dayton controller, product no. G0627006; Zoro) and set at maximum humidity. An air pump was connected to a sparger with a gas dispersion tube at the end, permitting small bubbles of air to escape and producing humidified air. Light sources were placed inside the chamber where appropriate and were connected to a power supply. Taking all the design features together, this chamber was largely sealed from the environment and its air was consistently humidified.

### Camera and software.

Images of microbial growth were captured using either a Canon Rebel XTi camera or a Nikon D3200 camera. To minimize the file size of the pictures captured, the lowest-resolution setting was chosen, resulting in images ranging from 400 to 600 kB each. The camera focus was set to manual to avoid automatic changes by the camera, which can result in flickering of images.

Remote control of the camera was achieved using the Canon-supplied EOS Utility software or a shutter cable release (Aputure timer camera remote shutter cable 3N). The camera was set to capture a picture every 10 min. Images were stored on an external server. Captured images were converted to a movie format using QuickTime Pro 7 software. Movies were made at a frame rate of 33 frames/s. Final movies were compressed using the open-source video transcoder Handbrake v1.0.1.

## Supplementary Material

Supplemental material

## References

[1] Hooke R 1665 Micrographia: or some physiological descriptions of minute bodies made by magnifying glasses, with observations and inquiries thereupon. Martin and Allestry, London, England [Reprint, Dover Publications, New York, NY, 1961.]

[2] van Leeuwenhoek A 1677 Letter of October 9, 1676, to the Royal Society. Philos Trans 12:821–831.

[3] Kaufmann R, Muller P, Hildenbrand G, Hausmann M, Cremer C 2011 Analysis of Her2/neu membrane protein clusters in different types of breast cancer cells using localization microscopy. J Microsc 242:46–54. doi:10.1111/j.1365-2818.2010.03436.x. 21118230

[4] Traxler MF, Watrous JD, Alexandrov T, Dorrestein PC, Kolter R 2013 Interspecies interactions stimulate diversification of the *Streptomyces coelicolor* secreted metabolome. mBio 4:e00459-13. doi:10.1128/mBio.00459-13. 23963177 PMC3747584

[5] Baym M, Lieberman TD, Kelsic ED, Chait R, Gross R, Yelin I, Kishony R 2016 Spatiotemporal microbial evolution on antibiotic landscapes. Science 353:1147–1151. doi:10.1126/science.aag0822. 27609891 PMC5534434

[6] Blanchard AE, Lu T 2015 Bacterial social interactions drive the emergence of differential spatial colony structures. BMC Syst Biol 9:59. doi:10.1186/s12918-015-0188-5. 26377684 PMC4573487

[7] Kobayashi K 2007 *Bacillus subtilis* pellicle formation proceeds through genetically defined morphological changes. J Bacteriol 189:4920–4931. doi:10.1128/JB.00157-07. 17468240 PMC1913431

[8] Holscher T, Bartels B, Lin YC, Gallegos-Monterrosa R, Price-Whelan A, Kolter R, Dietrich LE, Kovacs AT 2015 Motility, chemotaxis and aerotaxis contribute to competitiveness during bacterial pellicle biofilm development. J Mol Biol 427:3695–3708. doi:10.1016/j.jmb.2015.06.014. 26122431 PMC4804472

[9] Branda SS, Gonzalez-Pastor JE, Ben-Yehuda S, Losick R, Kolter R 2001 Fruiting body formation by *Bacillus subtilis*. Proc Natl Acad Sci U S A 98:11621–11626. doi:10.1073/pnas.191384198. 11572999 PMC58779

[10] Zafra O, Lamprecht-Grandio M, de Figueras CG, Gonzalez-Pastor JE 2012 Extracellular DNA release by undomesticated *Bacillus subtilis* is regulated by early competence. PLoS One 7:e48716. doi:10.1371/journal.pone.0048716. 23133654 PMC3487849

[11] Shapiro JA 1995 The significances of bacterial colony patterns. Bioessays 17:597–607. doi:10.1002/bies.950170706. 7646482

[12] Shapiro JA 1992 Pattern and control in bacterial colony development. Sci Prog 76:399–424. 1364579

[13] Vallotton P 2013 Size matters: filamentous bacteria drive interstitial vortex formation and colony expansion in *Paenibacillus vortex*. Cytometry A 83:1105–1112. doi:10.1002/cyto.a.22354. 24105998

[14] Ingham CJ, Ben Jacob E 2008 Swarming and complex pattern formation in *Paenibacillus vortex* studied by imaging and tracking cells. BMC Microbiol 8:36. doi:10.1186/1471-2180-8-36. 18298829 PMC2268691

[15] van Gestel J, Vlamakis H, Kolter R 2015 From cell differentiation to cell collectives: *Bacillus subtilis* uses division of labor to migrate. PLoS Biol 13:e1002141. doi:10.1371/journal.pbio.1002141. 25894589 PMC4403855

[16] Federation of American Societies for Experimental Biology. 2015 2015 BioArt winners. http://faseb.org/Resources-for-the-Public/Scientific-Contests/BioArt/Past-Winners/2015-BioArt-Winners.aspx.

[17] Rull J, Prokopy RJ 2000 Attraction of apple maggot flies, *Rhagoletis pomonella* (Diptera: Tephritidae) of different physiological states to odour-baited traps in the presence and absence of food. Bull Entomol Res 90:77–88. doi:10.1017/S0007485300000742. 10948367

[18] Ng W 2015 Possible odour-mediated attraction of flies to *Bacillus subtilis* NRS-762 stationary phase culture. PeerJ PrePrints 3:e541v3.

