# Supplementary material for: Visualizing Bacterial Colony Morphologies Using Time-Lapse Imaging Chamber MOCHA
Source: J Bacteriol. 2017 Dec 20;200(2):e00413-17. doi: 10.1128/JB.00413-17 (PMC5738739; doi:10.1128/JB.00413-17)
Supplement: Supplemental material [file supp_200_2_e00413-17__index.html]

Supplemental material 

# Visualizing Bacterial Colony Morphologies Using Time-Lapse Imaging Chamber MOCHA

## Supplemental material

- Supplemental file 1 -

  Movie S1 (Growth of bacillus *Arthrobacter agilis* and *Nesternkonia* colonies representing a blooming tree)

  MOV, 11M
- Supplemental file 2 -

  Movie S2 (*Bacillus*-*Serratia* interaction)

  MOV, 11M
- Supplemental file 3 -

  Movie S3 (Formation of *Bacillus* pellicle in a well)

  MOV, 16M
- Supplemental file 4 -

  Movie S4 (Formation of *Bacillus* pellicle in a beaker)

  MOV, 3.0M
- Supplemental file 5 -

  Movie S5 (Growth of a *Bacillus* colony)

  MOV, 5.6M
- Supplemental file 6 -

  Movie S6 (Growth of a fungus colony)

  MOV, 8.4M
- Supplemental file 7 -

  Movie S7 (*Bacillus* novel colony phenotype)

  MOV, 14M
- Supplemental file 8 -

  Movie S8 (Interaction of various *Streptomyces* strains)

  MOV, 9.3M
- Supplemental file 9 -

  Fig. S1 (Identical microbes grown in MOCHA) and S2 (Microbial imprint of kiss)

  PDF, 4.2M
- Supplemental file 10 -

  Movie S9 (Bacterial colony color development)

  MP4, 1.6M
