# Supplementary material for: Visualizing Bacterial Colony Morphologies Using Time-Lapse Imaging Chamber MOCHA
Source: J Bacteriol. 2017 Dec 20;200(2):e00413-17. doi: 10.1128/JB.00413-17 (PMC5738739; doi:10.1128/JB.00413-17)
Supplement: Supplemental material [file JB.00413-17_zjb002184619s9.pdf]

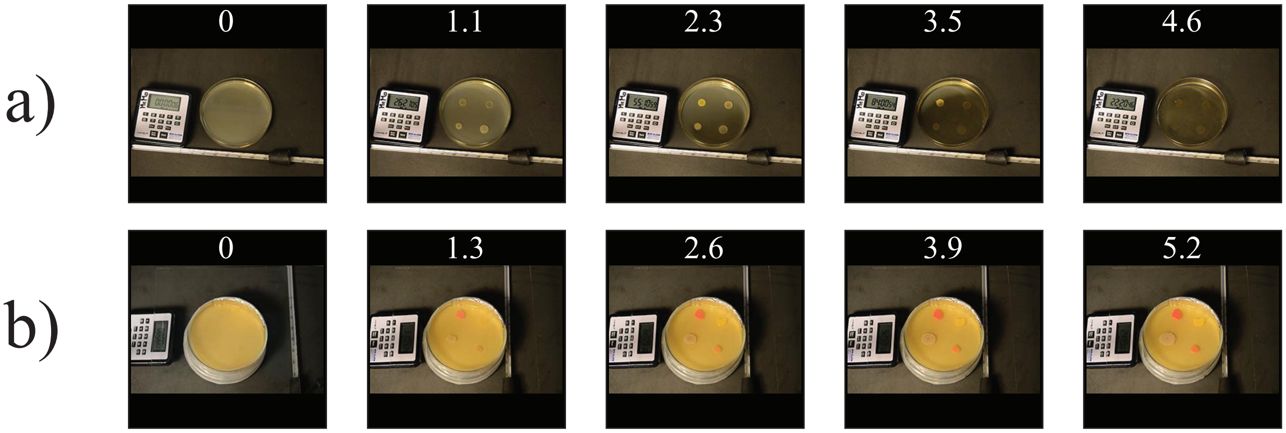

Supplementary Figure 1: Photographs of two time-lapse movies of identical microbes grown in MOCHA over time. Strains of (clockwise starting from top left) *Arthorobacter agilis, Nesternkonia spp, Bacillus pumilus* and *Deinococcus radiodurans* were innoculated wither (a) on a standard petri dish or (b) on a double-decker chamber and covered with museumglass. Time is shown in number of days above each photograph.

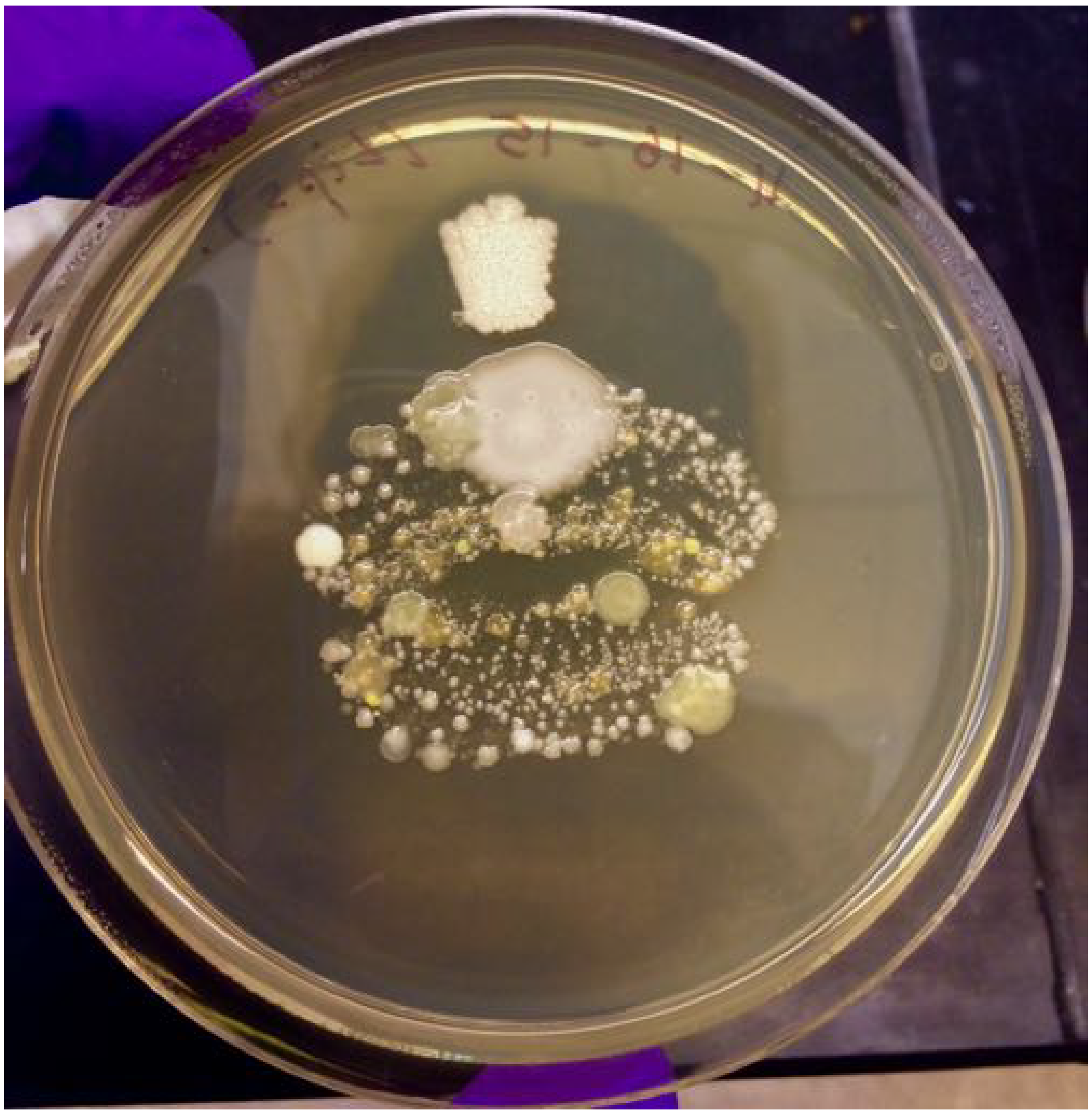

Supplementary Figure 2: Photograph of an agar plate showing the microbial imprint of the kiss of the artist Maria Peñil. .
